# Heterologous expression of anti-apoptotic human 14-3-3β/α enhances iron-mediated programmed cell death in yeast

**DOI:** 10.1371/journal.pone.0184151

**Published:** 2017-08-30

**Authors:** Rawan Eid, David R. Zhou, Nagla T. T. Arab, Eric Boucher, Paul G. Young, Craig A. Mandato, Michael T. Greenwood

**Affiliations:** 1 Department of Chemistry and Chemical Engineering, Royal Military College, Kingston, Ontario, Canada; 2 Department of Biology, Queen's University, Kingston, Ontario, Canada; 3 Department of Anatomy and Cell Biology, McGill University, Montreal, Quebec, Canada; Toho Daigaku, JAPAN

## Abstract

The induction of Programmed Cell Death (PCD) requires the activation of complex responses involving the interplay of a variety of different cellular proteins, pathways, and processes. Uncovering the mechanisms regulating PCD requires an understanding of the different processes that both positively and negatively regulate cell death. Here we have examined the response of normal as well as PCD resistant yeast cells to different PCD inducing stresses. As expected cells expressing the pro-survival human 14-3-3β/α sequence show increased resistance to numerous stresses including copper and rapamycin. In contrast, other stresses including iron were more lethal in PCD resistant 14-3-3β/α expressing cells. The increased sensitivity to PCD was not iron and 14-3-3β/α specific since it was also observed with other stresses (hydroxyurea and zinc) and other pro-survival sequences (human TC-1 and H-ferritin). Although microscopical examination revealed little differences in morphology with iron or copper stresses, cells undergoing PCD in response to high levels of prolonged copper treatment were reduced in size. This supports the interaction some forms of PCD have with the mechanisms regulating cell growth. Analysis of iron-mediated effects in yeast mutant strains lacking key regulators suggests that a functional vacuole is required to mediate the synergistic effects of iron and 14-3-3β/α on yeast PCD. Finally, mild sub-lethal levels of copper were found to attenuate the observed inhibitory effects of iron. Taken together, we propose a model in which a subset of stresses like iron induces a complex process that requires the cross-talk of two different PCD inducing pathways.

## Introduction

Cell death has been observed to occur in response to a variety of different conditions [1]. Classically, cellular death is separated into two basic forms, namely accidental and genetically encoded cellular suicide [2,3]. Accidental death, called necrosis, usually occurs in response to extreme stress and is not under the control of the cell. On the other hand, genetically encoded cell death occurs when a signaling and/or biochemical pathway is initiated resulting in the activation of cellular processes that lead to death via controlled cell destruction [2]. Historically, the term apoptosis was coined to describe all forms of genetically encoded cell death [4]. Early on, a separate form of programmed cell death that was associated with the accumulation of large vesicles, autophagosomes, was identified as being different from apoptosis and was called autophagy or type II PCD [5,6]. PCD has been and continues to be extensively investigated in part because it is disregulated in almost all human pathophysiologies [7–9]. One of the consequences of these extensive investigations is the identification of a large number and diversity of pathways that can serve to induce PCD. These multiple forms of PCD are associated with equally diversified cell death-inducing stimuli. Examples of such diversity include the induction of anoikis cell death in response to cellular detachment or the cell death called mitotic catastrophe that occurs when mitosis is interrupted [10,11]. More recently, a genetically encoded form of cell death resembling necrosis, called necroptosis, has been identified [12]. Stress-mediated PCD called intrinsic PCD, typically involves the activation of pro-apoptotic cascades mediated by the mitochondria [13–15]. This process includes the activation of central pro-apoptotic regulators like Bax, damage to mitochondria, accumulation pro-apoptotic second messengers like ROS, ceramide, and iron as well as the activation of molecules such as proteases (i.e. caspases) and nucleases that actually disintegrate the cell [2,14,16]. Understanding the mechanisms as well as the processes that get activated in response to the different stresses has served to increase our understanding of PCD. For example, caspase 8 has been identified as being important for the activation of the extrinsic apoptotic response following the stimulation of death receptor by for instance Tumor Necrosis Factors (TNFα) [17].

A number of mechanisms exist that serve to negatively regulate PCD. Cellular process such as autophagy, misfolded protein degradation by the proteasome, or the activation of the ER stress response by misfolded proteins are well known pro-survival processes that limit stress mediated PCD [2,7,18,19]. Also very commonly observed is the up-regulation of anti-apoptotic or pro-survival genes [2,20]. Many of these sequences encode proteins that directly interfere with pro-apoptotic proteins, for example, Bcl-2 is a strong inhibitor of PCD by binding to and preventing the action of the pro-apoptotic mediator Bax [21]. Similarly, Inhibitors of Apoptosis (IAP) function by preventing the actions of caspases [22]. Pro-survival sequences that function to decrease the levels of intracellular pro-apoptotic second messengers are also commonly described [2,23]. These include Super Oxide Dismutase (SOD) that decrease the levels of free radicals and Sphingomyelin Synthase (SMS1) that decreases the levels of ceramide [2,24]. A number of proteins that have chaperone activity like Heat Shock Protein 70 (HSP70) serve to prevent stress-mediated cell death by assisting in the refolding of damaged or denatured proteins [25]. A surprisingly large number of functionally unknown proteins also have pro-survival functions [2]. The characterization of many pro-survival proteins, for example, the iron storage protein ferritin, has increased our knowledge of the importance of iron in PCD. Alternatively, the study of cells expressing anti-apoptotic proteins has served to uncover different PCD pathways as well as the functional cross-talk between that exists between different pathways [2,3]. Thus the inhibition of caspase 8 and apoptosis by overexpressing IAPs in cells stimulated with death receptor agonists leads to the activation of alternative necroptotic pathways [12,26]. Thus caspase 8 is thought to function as a possible inhibitor of necroptosis. Similar switching of cell death modalities from apoptotic to necroptosis in response to apoptotic inducing stress has been observed in cells lacking the pro-apoptotic regulator Bax [27].

The genetically tractable yeast *S*. *cerevisiae* has proven to be an exceptional model to study basic cellular processes including PCD [10,28]. Yeast cells will undergo PCD in response to the same stimuli and stresses that induce PCD in mammalian cells [14,29]. Yeast stress mediated PCD is centered on the mitochondria and involves the activation of key apoptotic regulators including a metacaspase and a Bh3 containing protein [13,14,30]. Finally, a number of key processes such as increases in ROS and externalization of phosphatidylserine (PS) also occur in yeast as in mammalian apoptosis. Thus apoptosis, necrosis/necroptosis, autophagic as well as other cell death modalities are being investigated in yeast [2,14,31,32]. A variety of original and novel observations regarding the basic processes of PCD are being uncovered using yeast. For example, the central role of acetyl-CoenzymeA in modulating lifespan was first identified in yeast [33]. We and others have used yeast as a screening platform to identify and characterize cDNA sequences that can prevent yeast cells from undergoing stress mediated PCD [34,35]. In this way, we identified human 14-3-3β/α as a potent inhibitor of multiple stresses in yeast [35–37]. Yeast cells expressing 14-3-3β/α were found to be not only resistant to apoptosis but also to the cell death that occurs following prolonged autophagy inducing stimuli [36].

Here we examined the effect of different stresses on yeast cells expressing the pro-survival sequence human 14-3-3β/α [36]. As expected, these cells showed increased resistance to numerous PCD inducing stresses including copper and rapamycin. In contrast, 14-3-3β/α expressing cells showed a different response when challenged with other PCD inducing stresses including iron, hydroxyurea and zinc. In effect, 14-3-3β/α expressing cells were found to be hyperesponsive to the growth inhibitory and PCD inducing effects of iron. Analysis of mutants defective in genes involved in PCD responses revealed that the effect of iron required a functional vacuole. Taken together our results suggest that yeast activates at least two distinct pathways in response to some PCD stresses including iron.

## Materials and methods

### Yeast strains, plasmids, and growth

The *Saccharomyces cerevisiae* strain BY4742 (MATα *his3Δ1 leu2Δ0 lys15Δ0 ura3Δ0*) was used as the wild-type strain. All mutants *YCA1Δ*, *YBH3Δ*, *ATG1Δ*, *VMA3Δ* used in this study were isogenic to BY4742 and were obtained from ThermoScientific. Plasmids containing the cDNAs for 14-3-3β/α, TC-1, and FTH1 (human ferritin) expressed under the control of the galactose-inducible *GAL1* promoter in pYES-DEST52 (URA3 selectable marker) were isolated in our previously described Bax screen [35]. The plasmids expressing the cDNAs for yeast *YCA1* expressed under the control of the *GAL1*-galactose-inducible promoter were previously isolated and described [13,35,38]. The yeast media used in this study consists of yeast nitrogen base (YNB) and 2% glucose and the required amino acids (leucine, lysine, uracil and histidine). Yeast transformation was achieved by lithium acetate method and selected by omission of the appropriate nutrient. In order to induce the expression of sequences under the control of the *GAL1* promoter, 2% galactose, and 1% raffinose were added instead of 2% glucose.

### Spot and viability assays

To assess the ability of growth for different transformants in various kind conditions, spot assay was used [37]. Freshly glucose-grown saturated cultures of the different yeast transformants were diluted in fresh galactose media, incubated for 4–6 hours with shaking at 30°C, serially diluted ten fold with sterile water, and 5 μL of each dilution was spotted onto nutrient agar media followed by 3 to 4 days incubation at 30°C. The desired concentration of chemical stresses including copper sulfate (CuSO_4_) and iron chloride (FeCl_3_) were added directly to the media before pouring the plates as indicated in the figures. A concentration of 2.0 mM copper was used as the minimum concentration required to prevent the growth of wild type cells containing empty vector while 1.6 mM copper was used as the maximum concentration of copper that had minimal effect on growth of the control wild type cell [37]. A lower concentration of copper (0.5mM) was used to assess the growth of the *VMA3Δ* mutant. Iron was used at 6mM to assess its effect on the growth of wild type cells. In contrast, a higher concentration of iron (7mM) was used to assess the ability of sub-lethal copper to prevent the inhibitory effects of iron. A lower concentration of iron (2mM) was used to assess its effects on the growth of the *VMA3Δ* mutant. A minimum of three different spot assays with similar results were obtained. Viability was assessed by microscopic examination of cells stained with vital dye trypan blue, freshly inoculated galactose-growing cultures of the different transformants were challenged with copper sulfate (CuSO_4_) 1.4mM or iron chloride (FeCl_3_) 6mM and allowed to grow at 30°C for 18 hours. At least three different samples of 100 cells were scored for each experiment, and the experiments were repeated at least three independent times obtaining similar results. The data from the viability experiments are presented as the mean ± standard deviations of triplicate experiments repeated a minimum of three independent times. Statistical significance of the data was determined using a Student t-test. All described experiments were carried out a minimum of three independent times.

### Iron assay

Cells were inoculated at low or moderate cell densities in fresh galactose media and the cultures were grown for 18 hours with or without the addition of 10X more iron (10μM) than standard YNB media (1μM). Cells were harvested and washed three times with cold 1mM EDTA, flash frozen and stored at -80°C. The cells were broken with 3% nitric acid (98°C, 18hours) and iron content was determined using a colorimetric assay using the iron chelator ferrozine [37,39]. All described experiments were carried out a minimum of three independent times.

## Results

### Human 14-3-3β/α suppresses copper and enhances iron-mediated cell death

**Fig 1:**
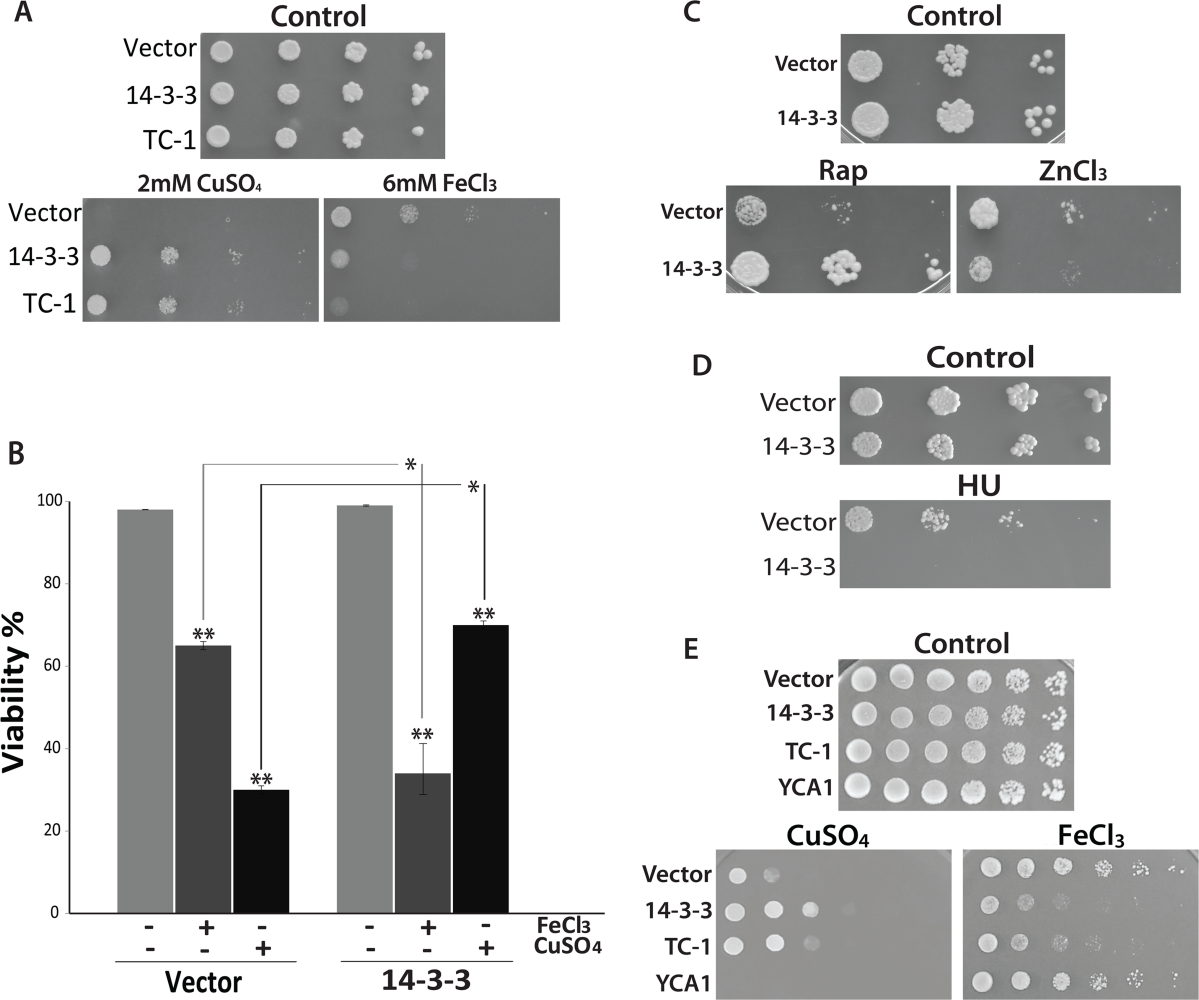
The effects of iron, copper, and HU on cells expressing the anti-apoptotic sequence 14-3-3. (A) Spot assays were used to assess the ability of wild-type cells expressing 14-3-3, and TC-1 to grow when challenged with 2mM copper (CuSO_4_) or 6mM iron (FeCl_3_). (B) The viability of cells containing empty vector and 14-3-3 expressing strains untreated (-) or treated (+) with 1.4mM copper (CuSO_4_) or 6mM iron (FeCl_3_) was determined by examining cells stained with trypan blue. Viability is shown as the mean percentage (%) of the cells that survived after treatment in triplicate experiments that were repeated at least 3 times.*; indicates significant differences between control cells (Vector) cells expressing 14-3-3 that were treated with copper or iron (p <0.001). **; indicates significant differences between control cells (not treated) and cells treated with copper or iron (p <0.001). (C) Spot assay was used to examine cell growth ability of the wild-type cells expressing 14-3-3 on nutrient agar containing 200nM rapamycin (Rap) or 20mM zinc chloride (ZnCl_2_). (D) Spot assay using nutrient agar containing 200mM Hydroxyurea (HU) was used to assess the growth of cells expressing 14-3-3. (E) Yeast cells harboring empty vector, as well as vector expressing 14-3-3, TC-1 and *YCA1* were serially diluted and spotted on YNB media with galactose alone (Control) or with 1.8mM copper (CuSO_4_) or with 5mM iron (FeCl_3_). The cells were incubated at 30°C to grow for 3 days.

Our lab has identified a number of pro-survival sequences by a direct screening of mammalian cDNA expression libraries in yeast cells undergoing PCD due to the expression of the pro-apoptotic Bax [35]. Although we confirmed the pro-survival function of many clones using stresses like copper, we did not fully explore the possibility that a specific sequence can selectively inhibit different PCD subroutines. One way to approach this is to determine if human 14-3-3β/α expression can prevent PCD in response to various stresses known to induce a different kind of cell death. Iron is an interesting compound for the study of PCD [40–42]. It is both an essential element as well as being highly toxic at supraphysiological levels [40–42]. In addition, excess intracellular iron is known to induce the production of highly toxic ROS species via the Fenton reaction [41]. Iron leads to DNA damage and this may contribute to the initiation of necrosis/necroptosis [43,44]. We were interested in determining the effect of iron on yeast cells expressing the previously characterized human pro-survival sequences 14-3-3β/α [36,38]. Using a spot assay, cells heterologously expressing 14-3-3β/α show the same growth as control cells containing empty vector when grown on agar media containing galactose alone with no additional stress (Fig 1A). Cells expressing 14-3-3β/α show more growth on media containing the lethal levels of copper when compared to control cells with vector alone (Fig 1A). This indicates, as previously described, that 14-3-3β/α is a pro-survival sequence capable of preventing PCD in response to stress [36]. When challenged with iron, the growth of the cells harboring 14-3-3β/α was noticeably inhibited compared to the control cells containing empty vector (Fig 1A). This suggests that heterologously expressed 14-3-3β/α increases the sensitivity of cells to the effects of iron. To determine if the observed effects with iron is a specific function of 14-3-3β/α, we examined TC-1 another previously characterized Bax suppressor that has been shown to attenuate the lethal effects of copper [38]. As with 14-3-3β/α, cells expressing TC-1 are more resistant to copper and more sensitive to iron (Fig 1A). This suggests that the observed iron effect is not specific to 14-3-3β/α.

**Fig 2:**
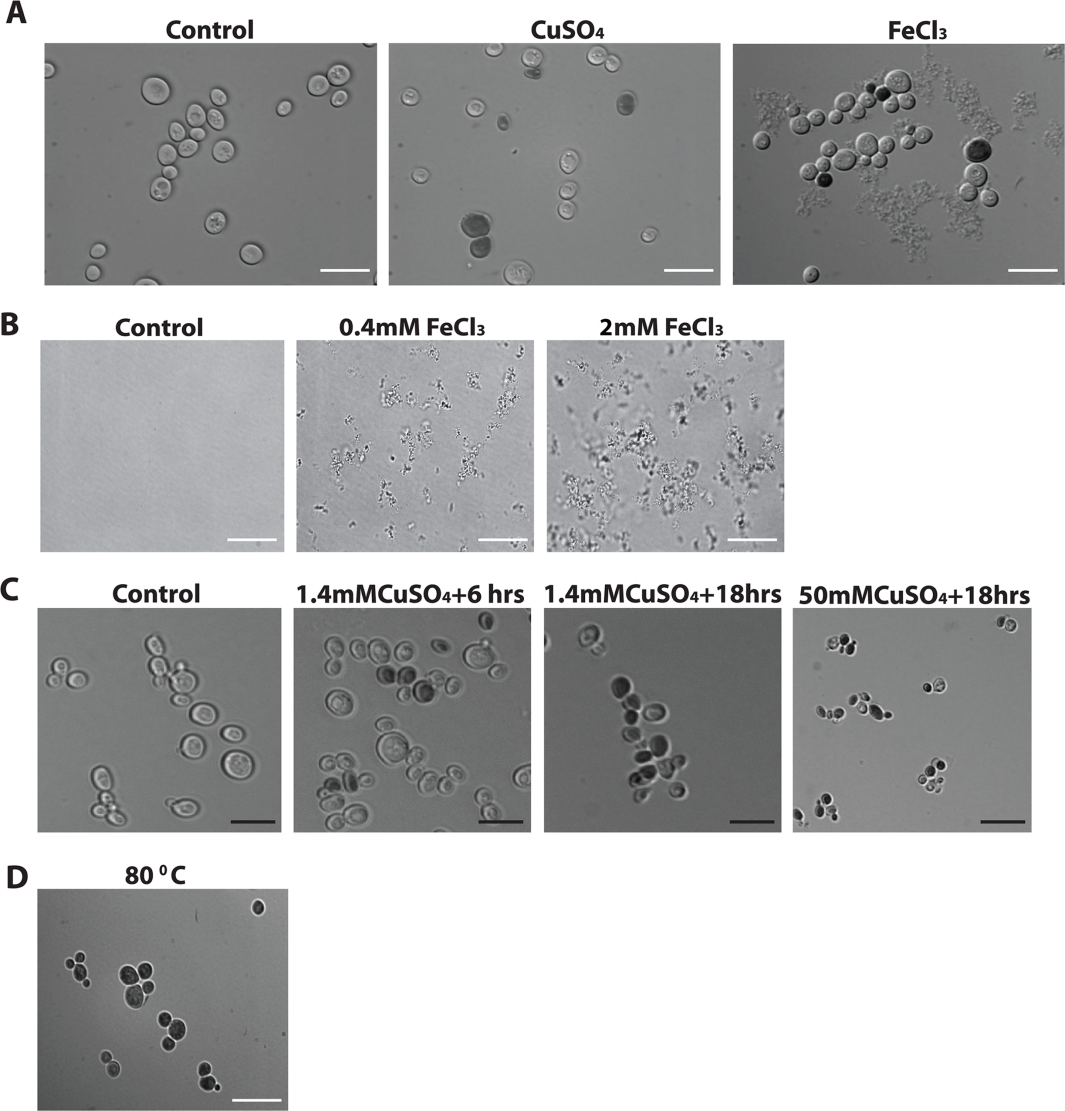
The effect of iron and copper stress on cell morphology. Yeast cells were grown in glucose for 24 hrs then inoculated in galactose media for 4 hrs followed by the addition of a different stress as explained in A,B and C. (A) Control cells or cells were inoculated with 1.4mM copper (CuSO_4_) or with 1mM iron (FeCl_3_) and their morphology were examined following treatment with trypan blue under the microscope. Live cells treated with trypan blue remained clear and appeared blue when dead. (B) Different concentrations of iron (FeCl_3_) were added to the media in the absence of cells. Media without iron (Control) is clear while with additive iron concentrations, 0.4mM FeCl_3_ and 2mM FeCl_3_, precipitation of iron is increased. (C) Yeast cells were non-treated or treated with various intensity of copper concentrations and different exposure time and examined under the microscope after staining with the vital dye trypan blue. Cells were treated with 1.4mM copper (CuSO_4_) for 6hrs, or for 18 hrs or 50mM copper (CuSO_4_) for 18hrs. (D) Cells were incubated at 80°C for 20min. and examined after inoculating with trypan blue. White scale bar, 100μm (A, D) and black scale bar, 20μm (B).

Because the spot assay measures net growth, we used microscopical examination of cells stained with the vital dye trypan blue to directly monitor the lethality of iron. The viability of cells in liquid culture was determined after 18h of incubation with or without iron or copper. For all strains, the viabilities remained above 98% after 18 hours of growth in the absence of additional stress (Fig 1B). In contrast, the viability of control cells had a decreased viability of 65% ± 5% when grown with iron and 27% ± 4% when grown with copper (Fig 1B). As expected, the cells expressing 14-3-3β/α showed a higher level of viability (78% ± 2%) when grown with copper as compared to control cells grown with copper (Fig 2B) [37,45,46]. In contrast, the viability of cells expressing 14-3-3β/α was decreased significantly when compared to control cells treated with iron (34% ± 15% vs. 65% ± 5%) (Fig 1B). This suggests that the expression of anti-apoptotic pro-survival sequences like 14-3-3β/α serves to enhance the lethality of iron.

We have previously shown that 14-3-3β/α expressing cells are resistant not only to copper but also to a number of other stresses including rapamycin [36,45]. Iron may differ from these other stresses for a number of reasons such as its ability to damage DNA [47]. We thus examined the effect of zinc (ZnCl_2_) and hydroxyurea (HU) as a stress. HU is known for its ability to inhibit ribonucleotide reductase, reversibly arrest cells in S phase and induce DNA damage [48]. Zinc (Zn^+^) has been reported to interact with DNA, decrease mitochondrial membrane potential, and weaken lysosomal stability [49,50]. Using spot assays, cells expressing 14-3-3β/α showed similar growth as cells harbouring empty vector on nutrient agar media containing galactose alone (Fig 1C and 1D). When challenged with rapamycin (Rap), as expected, cells expressing 14-3-3β/α showed more growth than cells containing the empty vector (Fig 1C). In contrast, cells expressing 14-3-3β/α were noticeably more affected by zinc and HU than cells containing the empty vector (Fig 1C and 1D). This suggests that the anti-apoptotic sequence 14-3-3β/α is not specific only in enhancing the lethality of iron but also it does the same with zinc and HU. Taken together the results in this section suggests that overexpression of pro-survival sequences induce a cellular state that makes the cell resistant to the lethal effects of some stresses such as copper and rapamycin [36,38,45] while making the cell more sensitive to other stresses that include iron, zinc and HU.

Overexpressing or knocking out a gene and examining changes in phenotypes is a common genetic approach for exploring the function of individual genes in biological processes [51]. In spite of the many successes using these strategies, it remains that artifacts are known to occur with these approaches [52]. For example, global analysis of the entire repertoire of yeast genes revealed that many genes lead to a slow growth phenotype when overexpressed [52]. It seems likely that the slow growth phenotype observed with many sequences is the result of non-specific obstruction of cell processes and not due to a specific defect [51]. To address the possibility that the observed effects of 14-3-3β/α with iron may be non-specific, we generated yeast transformants that overexpress a different functional protein, namely the pro-apoptotic sequence *YCA1* (also called *MCA1*) [13]. Using the spot assay, we thus examined how yeast cells overexpressing *YCA1* differ in their response to copper and iron in comparison to control cells with empty vector. All four strains tested including empty vector control, as well as transformants harboring vectors for the expression of 14-3-3β/α, TC-1, and *YCA1* show similar growth on control galactose agar media alone (Fig 1E). Differential effects on growth are observed when the strains are spotted on galactose inducing media containing copper or iron. On plates containing copper, the growth of the vector-containing control strain is greatly reduced, and this inhibition is, as expected, attenuated in cells expressing 14-3-3β/α or TC-1 (Fig 1E). In contrast, the growth of cells overexpressing *YCA1* is more inhibited than the control strain (Fig 1E). The copper stress serves to activate the *YCA1* protein leading to increased PCD [45]. As shown above (Fig 1A) with iron stress, the growth of the 14-3-3β/α and TC-1 expressing strain is reduced compared to the vector containing strain (Fig 1E). In contrast, the growth of *YCA1* cells is noticeably less affected by the presence of iron compared to cells containing empty vector (Fig 1E). Thus overexpressed *YCA1* is a functionally expressed protein that can enhance the effects of copper and does not alter a cell’s response to iron. Taken together, the effects of 14-3-3β/α and TC-1 on iron treated cells appear to be specifically linked to their pro-survival effects.

### Morphology of iron and copper treated cells

In cultured mammalian cells, microscopical examination may serve to differentiate between different modes of cell death. For example, it is commonly observed that apoptotic cells remain intact during the early process of cell death while cells lose all structure and seem to explode early on during necrosis/necroptosis [3,53]. In contrast, the distinction between apoptosis and necrosis/necroptosis is not easily determined in yeast. It is known that apoptotic-like cell death can be induced in yeast by a variety of different stresses including acetic acid, copper, H_2_O_2,_ and cycloheximide [36–38,45,54–56]. Necroptosis/necrosis cell death as well as the cell death that occurs in unstressed wild type cells in response to extracellular iron occurs in yeast but these processes have been much less studied [31,57]. A number of stresses, including iron, have been reported to favor the induction of necroptosis over other forms of PCD [31,43,47]. Thus we microscopically examined cells stained with the vital dye trypan blue in order to examine the morphology of both viable and inviable yeast cells treated with 1.4mM copper or 1mM iron (Fig 2A). It should be noted that 1mM represents a 1000-fold excess over the normal 1μM iron present in yeast YNB growth media [44]. As shown, two distinct cell types could be identified, namely clear live cells as well as dead cells that are blue (Fig 2A). In addition, we also observed what appeared to be some sort of debris in the medium associated with cells grown with iron (Fig 2A). This debris is likely some sort of iron precipitate, since it can be observed to increase in a dose dependant manner when iron alone is added to the YNBD growth media (Fig 2B). The tendency of iron to precipitate with time, altered pH or in mixed solutions is one of the major and common problems associated with matintaining iron at sufficiently high concentrations to induce cell death in normal unstressed cells [42,44].

It remained that we were still interested in determining if we could observe alterations in cell morphology with different stresses. An alternative way to induce necrosis/necroptosis is to increase the concentration or duration of a normally apoptotic inducing stress [54]. Thus we examined cells that were treated for 6 or 18hours with 1.4mM copper or 18hours with 50mM copper. Although there was a decrease in viability with the first two conditions (respectively 80%, 30%), there was no noticeable change in cellular morphology. Similarly, there were no observable necrotic-like morphology in cells treated with 50mM copper even though a more pronounced decrease in viability was observed (20%) (Fig 2C). Nevertheless, we do observe a decrease in cell size in these cells (Fig 2C). The decrease more than likely reflects that cell size is reduced in slower growing cells and that stressed cells reduce their growth rate prior to initiating PCD [58,59]. We also examined the morphology of yeast cells that were incubated at 80°C for 20 min. Following this treatment, all of the cells appeared blue indicating that they were all dead. In spite of this, all the cells appear to have maintained their intact cellular morphology (Fig 2D). Taken together the results here show that either iron can not induce a cell death that resembles necrosis or that necrotic yeast cells do not differ in their morphology compared to apoptotic cells.

### Analysis of 14-3-3β/α and iron in mutants defective in apoptosis, autophagy, and vacuolar function

**Fig 3:**
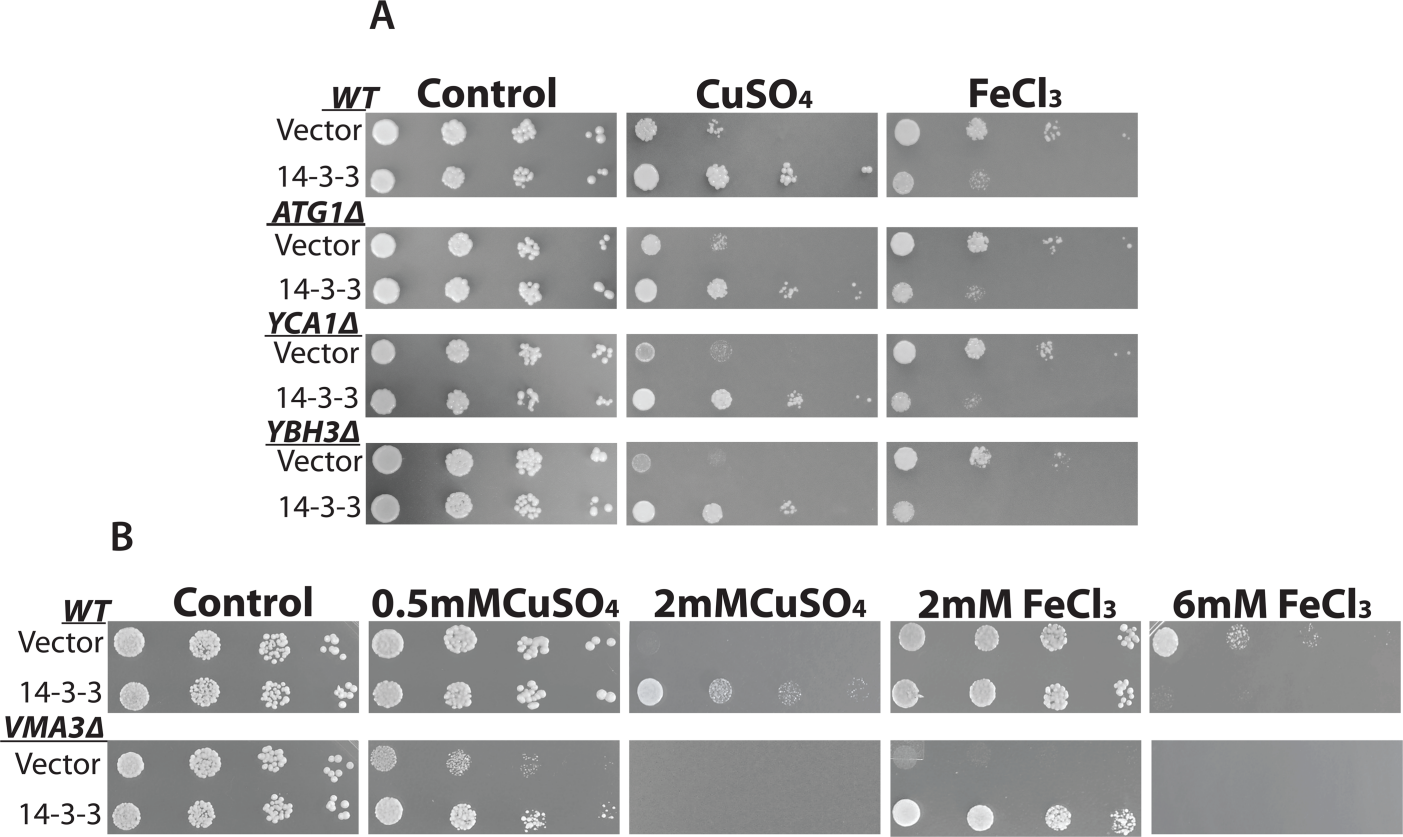
The effects of copper and iron on yeast mutant cells. Serially diluted cultures of WT and yeast mutants with empty vector and a plasmid expressing 14-3-3 were spotted on galactose media with and without copper (CuSO_4_) or iron (FeCl_3_). (A) The mutants used are *ATG1Δ* for autophagy, *YCA1Δ*, and *YBH3Δ* for apoptosis. The concentrations used are 2mM CuSO_4_ and 5.5mM FeCl_3_. (B) Mutant used is *VMA3Δ* for necrosis. The concentration of copper used is 0.5mM and 2mM CuSO4 and iron concentration is 2mM and 6mM FeCl_3_. The plates were incubated at 30°C for four days.

Identification of specific proteins involved in mediating the PCD inducing effects of different stresses can serve to characterize different PCD pathways [2,3]. For example, using genetic approaches, it has been found that the yeast metacaspase *YCA1p* is involved in mediating the effects of acetic acid but not copper [13,54]. Thus here we used mutants devoid of key genes involved in regulating PCD including *YCA1*, *YBH3* (a BH3 containing Bax-like protein) and *ATG1* (autophagy-defective) to examine their possible roles in mediating the effects of iron with 14-3-3β/α. The mutants were transformed with empty vector or 14-3-3β/α expressing plasmid and their growth in the presence of copper and iron were determined using the spot assay (Fig 3A). All the mutants showed growth inhibition by copper and iron. As shown previously (Fig 1A) [45], the expression of 14-3-3β/α was able to increase copper resistance as determined by increased growth for all three mutants examined (Fig 3A). As observed in wild-type cells, the expression of 14-3-3β/α served to enhance the inhibitory effect of iron in the three mutants examined. These results indicate *YCA1*, *YBH3*, and *ATG1* are not likely involved in mediating the effects of 14-3-3β/α on iron.

In yeast, the vacuole (lysosome in mammals) is critical for iron metabolism [60]. Thus many mutants defective in vacuolar function are hypersensitive to iron [37,60,61]. In addition, the vacuole/lysosome has been linked to the induction of necrosis/necroptosis [62]. Mutants defective in the vacuolar proton pump due to the loss of one of the many different genes that encode for the Vacuolar Membrane ATPase (*VMA*) show reduced vacuolar function, are more resistant to necroptosis and are hypersensitive to iron [61,63]. The mutant *VMA3Δ* as well as the wild-type strain were transformed with empty vector or the 14-3-3β/α expressing plasmid and their ability to grow with copper and iron evaluated using the spot assay (Fig 3B). The *VMA3Δ* mutant with and without 14-3-3β/α were able to grow like the wild strain on galactose-inducible media (Fig 3B). In contrast, the *VMA3Δ* mutant showed enhanced sensitivity to copper and iron when compared to wild-type cells (Fig 3B). Thus *VMA3Δ* cells with empty vector showed significantly reduced growth in the presence of 0.5mM copper or 2mM iron. In contrast, these concentrations of iron or copper did not affect the growth of wild-type cells (Fig 3B). Thus we evaluated the effects of copper and iron respectively at 0.5mM and 2mM. At these concentrations both copper and iron had no effect on the growth of wild-type cells (Fig 3B). In spite of the increased sensitivity, the expression of 14-3-3β/α served to promote the growth of *VMA3Δ* cells treated with copper or iron (Fig 3B). This is in contrast to the effects of iron, in wild-type cells where the expression of 14-3-3β/α enhances the inhibitory effects of iron (Fig 3B and also see Fig 1). In the mutants, the expression of 14-3-3β/α now serves to attenuate the inhibitory effects of iron (Fig 3B). This indicates that there is a difference in response to iron in the *VMA3Δ* mutant. These data not only confirm that a functional vacuole is required for resistance to multiple stresses including iron [61], but that it is also crucial for mediating the normal and specific PCD responses to high iron stress [37,60].

### Copper conditioning prevents iron inhibition

**Fig 4:**
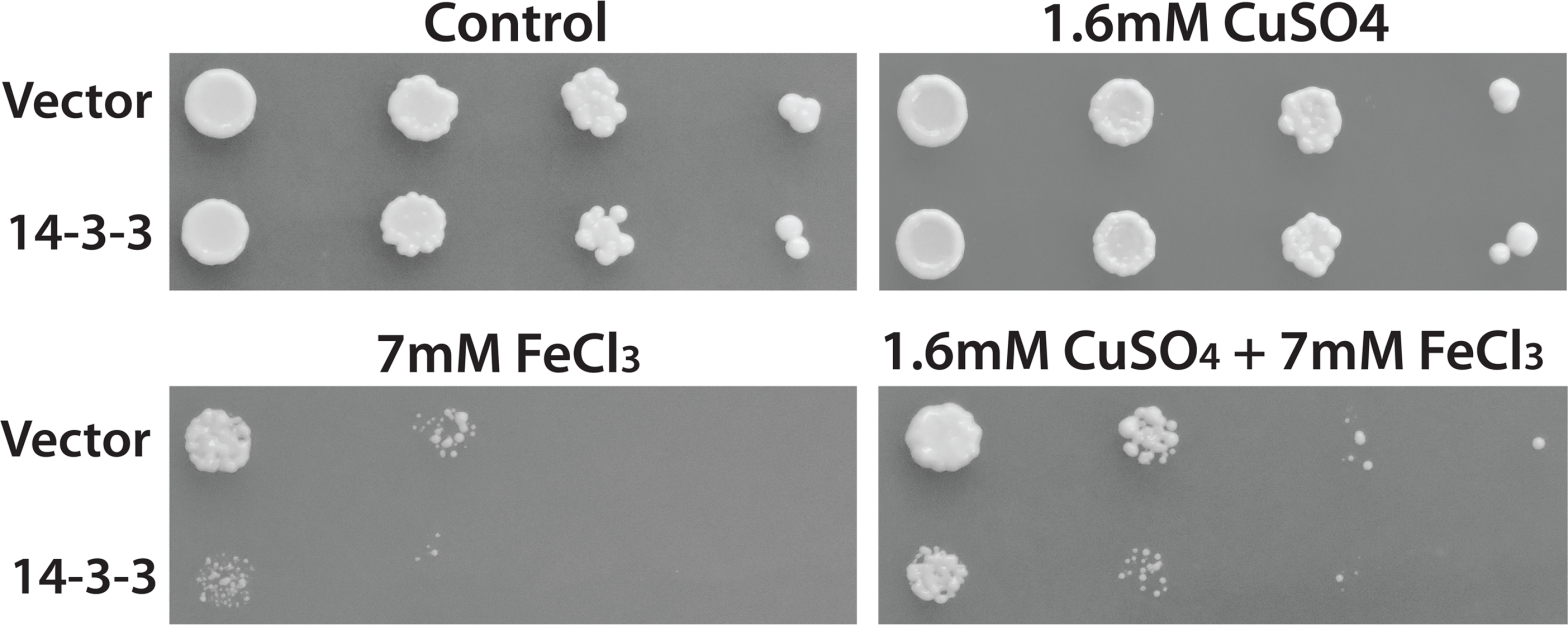
Sub-lethal copper conditioning inhibits iron-mediated 14-3-3 lethality. Yeast cells with empty vector or 14-3-3 expressing vector were serially diluted and spotted on nutrient agar plates containing galactose alone (Control) or with 1.6mM copper (CuSO_4_), 7mM iron (FeCl_3_), or with both1.6mM copper (CuSO_4_) and 7mM iron (FeCl_3_). Plates were incubated at 30°C for four days.

It is well known that there is significant cross talk between different PCD stress responsive pathways [2,18,64]. To further examine possible differences between the iron and copper responses we examined if there is cross- talk between the copper and iron-responsive pathways. To do this, we made use of the fact that sub-lethal levels of stresses are known to induce a protective state often called pre-conditioning or hormesis that increases resistance to stronger PCD inducing stresses [7,65]. The protective sub-lethal stress can be added not only prior to the lethal stress but also can be added after or at the same time of the lethal stress [66]. Thus we grew yeast transformants with sublethal copper in the presence and absence of lethal levels of iron. Freshly saturated cultures of control, as well as 14-3-3β/α expressing yeast transformants, were serially diluted and spotted onto nutrient agar media containing 1.6mM CuSO_4_, 7mM FeCl_3_ or both 1.6mM CuSO_4_ and 7mM FeCl_3_. There is no difference in the growth of cells grown on galactose media with or without the presence of 1.6mM CuSO_4_ in the media (Fig 4). In the presence of 7mM FeCl_3_, there was a significant decrease in the growth of cells containing the empty vector which was enhanced in cells expressing 14-3-3β/α (Fig 4). In contrast, the growth of both transformants in the presence of iron was enhanced by sub-lethal copper. This suggests that conditioning with sub-lethal copper reduces the lethal effects of iron and further support fundamental differences existing in processes mediated by copper and iron.

### Human ferritin protects against copper but not iron stress

**Fig 5:**
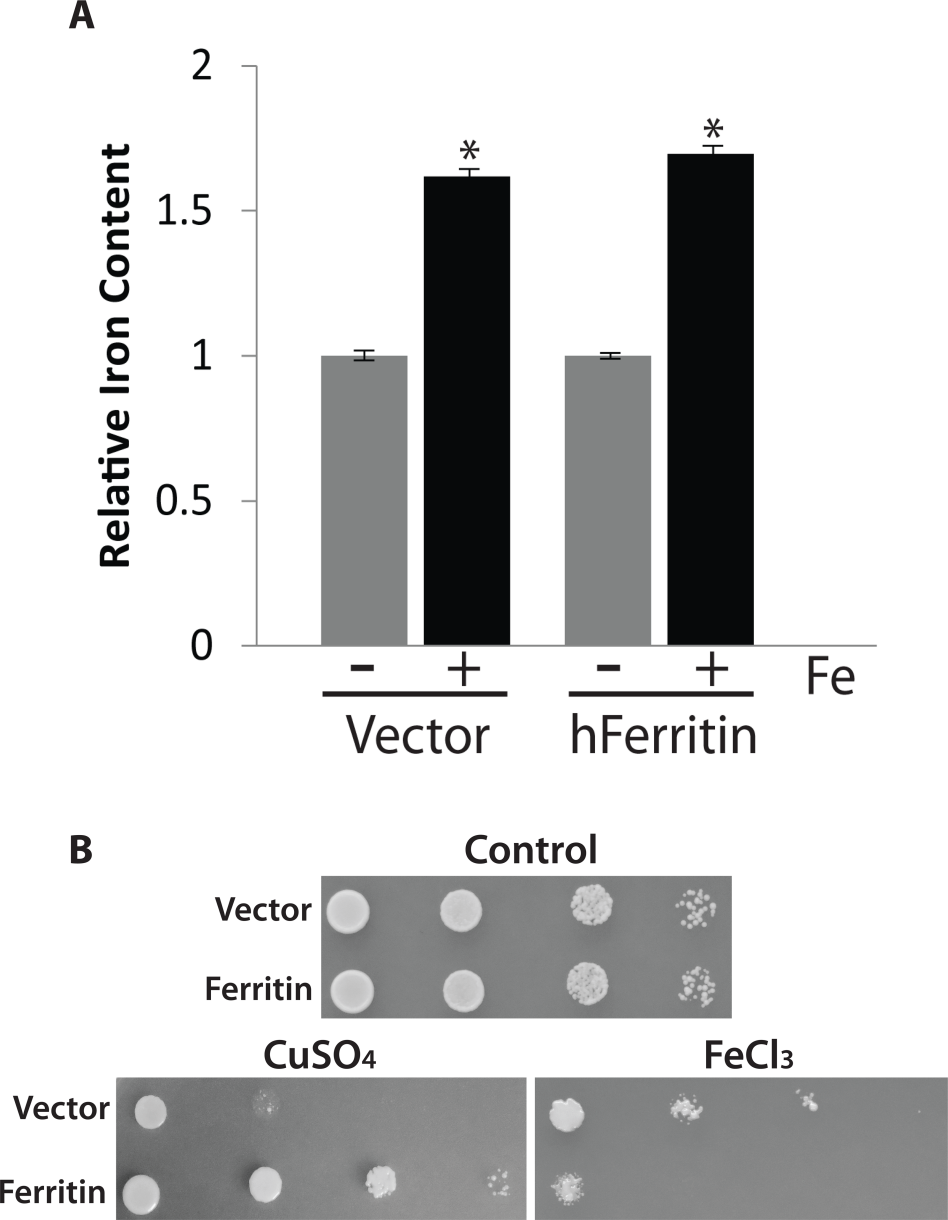
Ferritin enhances iron toxicity and doesn’t show enhanced iron storage in yeast. (A) Yeast cells with empty vector (Vector) or expressing ferritin were grown in YNB glucose for 24 hrs and then inoculated in YNB galactose for 18hrs in absence or presence of a 10 fold excess of iron (FeCl_3_). The cells were harvested, and total iron content was determined using a ferrozine-based colorimetric assay. Relative iron content found in yeast cells (Vector) growing in YNB galactose media (1 = 5.2 ± 0.4 ng iron/10^8^ cells). The mean of the triplicate experiment was calculated, and the experiment was repeated 3 times. *, indicates significant difference from the values obtained when grown in the absence and the presence of 10 fold excess iron (p <0.05). (B) Spot assay was used to assess the ability of yeast cells expressing ferritin to grow when spotted on YNB galactose alone (control) or with 2mM copper (CuSO_4_) or with 6mM iron (FeCl_3_). These plates were incubated at 30°C for 3 days.

Cell death mediated by extracellular stresses is a complex process that is in large part dictated by the nature of the stress used [7,64,67]. Many stress including compounds such as hydrogen peroxide are freely diffusible and cause stress by entering the cell, reacting with cellular constituents and producing free radicals. Others, such as iron and copper are not so simple. Although both these ions are essential micronutrients and are critical for cell growth they are also very toxic at elevated levels [44]. As a consequence, an increase in the intracellular levels of most micronutrients is limited by a large number of regulatory processes even when the extracellular concentration of a micronutrient is high [2,68]. We have documented this process here for iron. Normal minimal YNB growth media used for yeast contains ca. 1μM iron. We grew yeast cells in the presence of a 10-fold excess iron, a concentration where all the iron remains soluble [44]. As we have previously shown for wild type cells, the observed increase in total intracellular iron was limited to 60%, indicating that increases in intracellular iron are effectively limited (Fig 5A) [37,69]. For iron, the situation is even more complicated by the fact that many PCD inducing stresses, including extracellular iron, lead to increases in levels of free bioactive iron [44]. Thus, the fact that intracellular iron chelators can prevent stress mediated PCD strongly supports the notion that iron is a stress inducible intracellular pro-apoptotic second messenger much like ROS and ceramide [57]. Overexpression of mammalian ferritin is reported to increase total iron content in mammalian and in yeast cells and protect the cells from stress including excess iron [37,70,71]. We and others have previously shown that human ferritin can prevent iron mediated cell death but only in iron supersensitive mutants [37,71,72]. The ability to attenuate the effects of iron in wild type yeast has actually never been examined for ferritin. Here we show as expected from our previous studies, using spot assays, that wild type yeast cells that are expressing human ferritin are more resistant to the effects of copper (Fig 5B). In contrast the expression of ferritin renders wild type more sensitive to the negative effects of iron (Fig 5B). This is similar to what we observe with 14-3-3 which suggests that ferritin in yeast retains its pro-survival function. The measurement of the total iron content of ferritin expressing yeast cells grown with a 10-fold excess of iron did not differ from what we observe in control cells (Fig 5A). This suggests that ferritin cannot be used to manipulate total iron content in yeast. As an alternate method to quench and sequester excess intracellular iron and to decrease stress mediated PCD is to use cell permeable iron chelators [73]. Unfortunately the use of an iron chelator to study the role of intracellular iron in mediating the effect of extracellular iron are difficult and we were unable to show an effect of iron chelators with hydroxyurea (not shown).

## Discussion

In this study, we examined the ability of anti-apoptosis induced by the expression of human 14-3-3β/α to prevent cell death in response to different stresses. We hypothesized that different stresses would induce different forms of PCD that could be differentially inhibited by 14-3-3β/α. We had previously shown that 14-3-3β/α could inhibit PCD from multiple stresses including copper and rapamycin as well as block different processes including apoptosis and autophagic cell death as induced by prolonged leucine starvation [14,36–38,45]. Thus it was surprising to find that wild type cells expressing 14-3-3β/α showed an increased sensitivity to iron (Fig 1A and 1B). The ability to prevent iron-mediated cell death by inducing anti-apoptosis has surprisingly been little studied [74]. In fact, most of the studies that examine iron mediated programmed cell death induced by iron are themselves not studying iron alone as an inducer of apoptosis or other forms of PCD. This is in large part due to the rather low solubility of iron and the difficulty in maintaining concentrations of iron in the growth media that are sufficiently high to induce PCD (Fig 2B) [44]. To circumvent these problems, most studies have used sub-lethal iron concentrations in combination with other sub-lethal stresses [44,74,75]. For example, serum free medium is commonly used, but not commonly acknowledged, as a sub-lethal secondary stress that is absolutely required to induce iron mediated apoptosis [44]. With yeast, we and others have largely used yeast mutants that are supersensitive to iron in order to induce PCD that is characterized as being iron mediated [37,44]. Thus we were able to show that the expression of the pro-survival human ferritin could prevent iron mediated PCD in the iron sensitive *VMA3Δ* yeast strain but not in the wild type cell. Our results show that there is clearly a difference in studying iron mediated cell death in the presence or absence of any other secondary sub-lethal stress.

**Fig 6:**
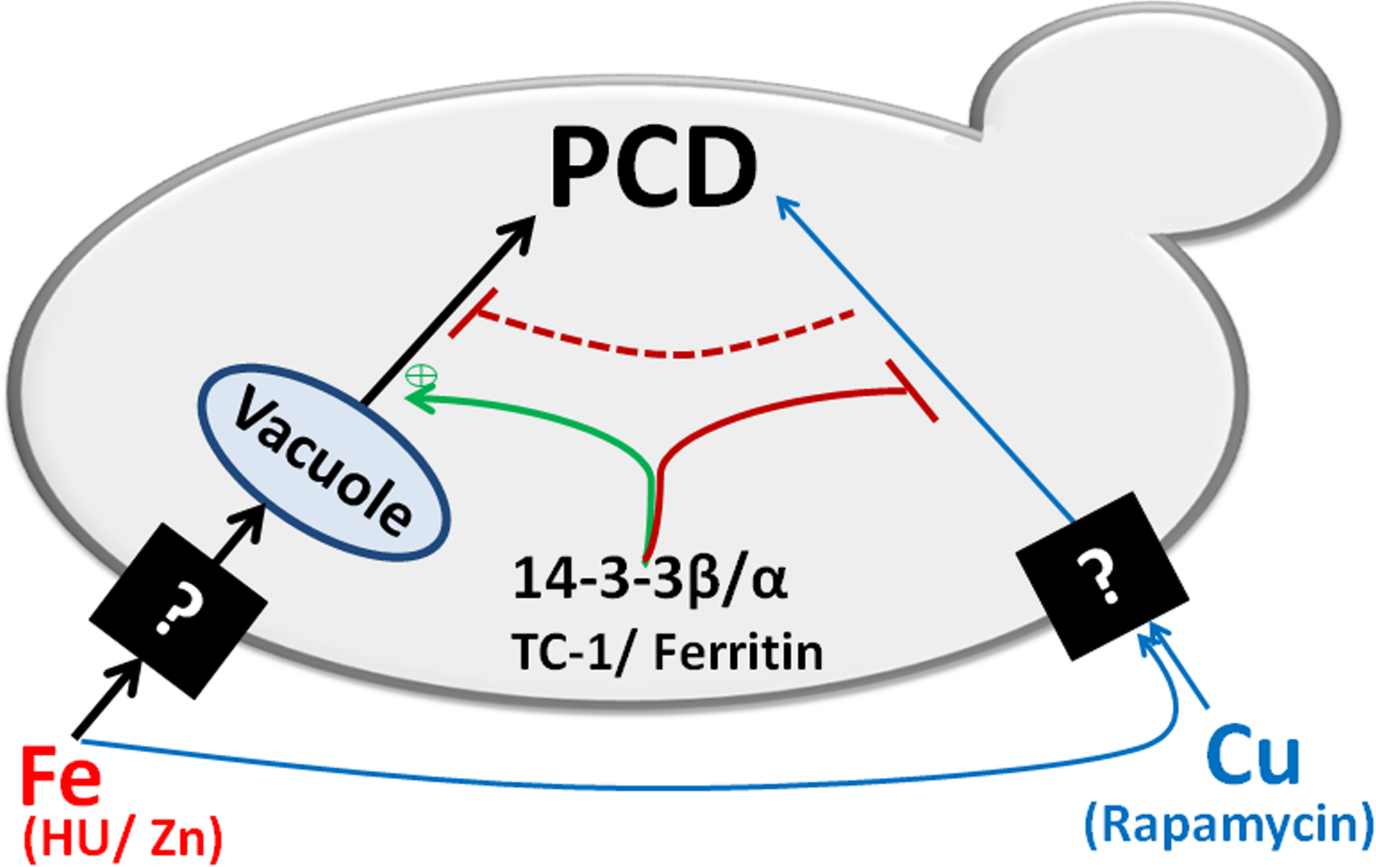
A model depicting the cross-talk between copper and iron mediated PCD in yeast. Exogenous copper (Cu) induces PCD in a single pathway while iron induces PCD using two different pathways. One of the iron-dependent pathways relies on the presence of a functional vacuole, and it is enhanced by anti-apoptotic genes including 14-3-3β/α, TC-1, and ferritin. While the other iron pathway is likely identical to the copper inducible PCD pathway since both can be inhibited by the anti-apoptotic sequences. This model showed that the two different pathways suppress each other and the anti-apoptotic sequences play an important role in this cross-talk.

Our main finding is that blocking PCD by expressing an anti-apoptotic sequence (14-3-3β/α) makes a cell super sensitive to the growth inhibitory as well as the cell death-inducing effects of iron (Fig 1). These results are reminiscent of the studies that have reported on the effect of blocking of apoptosis with caspase 8 inhibitors, in cells induced to undergo apoptosis by death receptor agonists [12,26]. Indeed, instead of activating the extrinsic apoptotic pathway, these cells were found to induce an alternate cell death pathway that resembles necroptosis [26]. A model in which death receptor simultaneoulsy activates both apoptosis and necroptosis appears to explain the observation [26]. In this model, caspase 8 would serve to simultaneously activate the apoptotic pathway and inhibit the necroptotic pathway. Blocking caspase 8 would thus prevent apoptosis and activate necroptosis. This type of cross-talk would then allow a cell more control over the process of PCD depending on the cellular context. Inspired by the work on caspase 8 we have developed a similar model that is based on the existence of two different but cross-talking PCD pathways in yeast (Fig 6). One of the pathways is activated by copper. This pathway leads to a programmed cell death that can be inhibited by the expression of 14-3-3β/α [37,38,76]. Iron differs since it activates the copper pathway all the while simultaneously activating a second pathway. The activated copper pathway would negatively regulate the iron pathway. This would explain why blocking the copper pathway by expressing 14-3-3β/α would enhance the lethal effects of iron. In cells where the iron specific pathway is blocked due to loss of vacuolar function (*VAM3Δ*), iron would activate only the copper pathway. This would explain why iron mediated PCD is blocked by 14-3-3β/α in the *VAM3Δ* cells. The vacuole (lysosome) has been shown to be involved in mediated the process of cell death [2,44,62,63]. During necrosis and necroptosis, the lysosome is thought to break open resulting in the spillage of catalytically active destructive proteins including proteases and RNAses as well as other destructive materials such as redox active iron that serve to help in the destruction of the cell. Thus cells with reduced vacuolar function are thought to be more resistant to the induction of necroptosis. Our model suggests that a functional vacuole is actively involved in mediating the cell death inducing responses to some stresses such as iron.

Finally, we exploited the fact that in all cells including yeast, exposure to sub-lethal stress can lead to both an increase in the resistance to a higher concentration of the same stress as well as an increase resistance to heterologous stresses [77–79]. We showed that wild type cells growing on media containing sub-lethal copper are more resistant to high levels of iron (Fig 4). These results further support the model by demonstrating that there is functional cross-talk between iron and copper pathways in yeast (Fig 6). We are currently testing this model by using chemical inhibitors of the vacuole as well as screening for genes that would prevent iron mediated cell death in cells expressing 14-3-3β/α.

In conclusion, our results suggests that there is a complex interplay between the processes that activate and prevent programmed cell death in response to different stresses in yeast.
